# Supplementary material for: Complex‐centric proteome profiling by SEC‐SWATH‐MS
Source: Mol Syst Biol. 2019 Jan 14;15(1):e8438. doi: 10.15252/msb.20188438 (PMC6346213; doi:10.15252/msb.20188438)
Supplement: Supplementary file 7 — Dataset EV6 [file MSB-15-e8438-s007.zip › feature_plots_bioplex/Q9Y5U2.pdf]

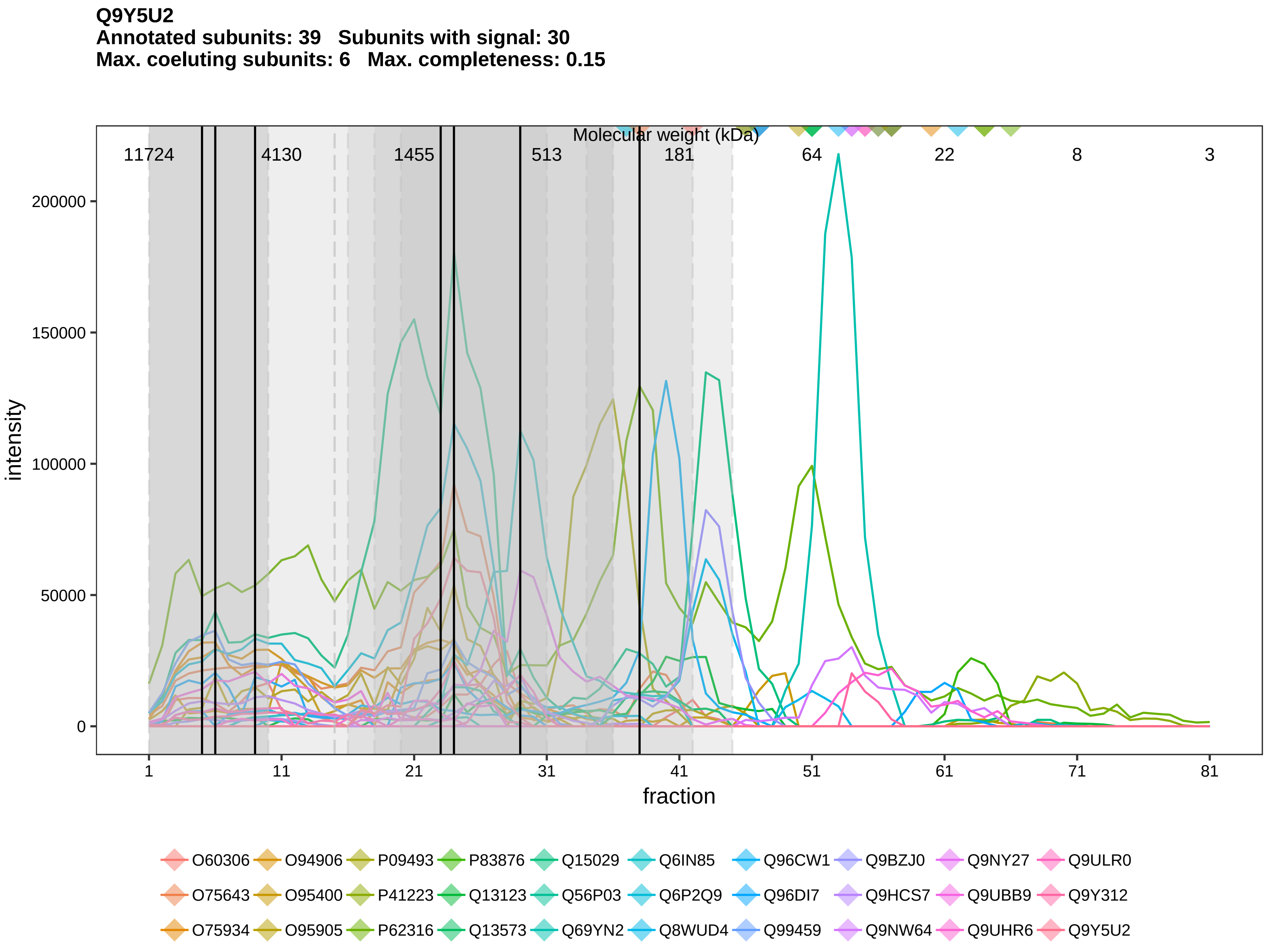
Q9Y5U2
Annotated subunits: 39 Subunits with signal: 30
Max. coeluting subunits: 6 Max. completeness: 0.15
Molecular weight (kDa)
11724
4130
1455
513
181
64
22
8
3
intensity
200000
150000
100000
50000
0
1
11
21
31
41
51
61
71
81
fraction
O60306
O94906
P09493
P83876
Q15029
Q6IN85
Q96CW1
Q9BZJ0
Q9NY27
Q9ULR0
O75643
O95400
P41223
Q13123
Q56P03
Q6P2Q9
Q96DI7
Q9HCS7
Q9UBB9
Q9Y312
O75934
O95905
P62316
Q13573
Q69YN2
Q8WUD4
Q99459
Q9NW64
Q9UHR6
Q9Y5U2
